# Pharmacokinetic analysis of two different docetaxel dose levels in patients with non-small cell lung cancer treated with docetaxel as monotherapy or with concurrent radiotherapy

**DOI:** 10.1186/1471-2407-7-197

**Published:** 2007-10-23

**Authors:** Paal Fr Brunsvig, Anders Andersen, Steinar Aamdal, Vessela Kristensen, Harald Olsen

**Affiliations:** 1 Department of Medical Oncology, Cancer Clinic, Rikshospitalet-Radiumhospitalet Medical Centre, Montebello, 0310 Oslo, Norway; 2 Department of Clinical Pharmacology, Laboratory Clinic, Rikshospitalet-Radiumhospitalet Medical Centre, Montebello, 0310 Oslo, Norway; 3 Department of Clinical Cancer Research, Cancer Clinic, Rikshospitalet-Radiumhospitalet Medical Centre, Montebello, 0310 Oslo, Norway and Faculty of Medicine, University of Oslo, Norway; 4 Department of Genetics, Institute for Cancer Research, Rikshospitalet-Radiumhospitalet Medical Centre, Montebello, 0310 Oslo, Norway

## Abstract

**Background:**

Previous pharmacokinetic studies with docetaxel have mostly used 3-weekly (75 mg/m^2 ^and 100 mg/m^2^) or weekly regimens (35–40 mg/m^2^). The pharmacokinetics and radiosensitizing efficacy of weekly 20 mg/m^2 ^docetaxel, has however not been well characterized. We examined the pharmacokinetics of weekly docetaxel when administered with concurrent radiotherapy and compared the results with a 3-weekly 100 mg/m^2 ^regimen.

**Methods:**

Thirty-four patients with non small cell lung cancer (NSCLC) were included in this study, 19 receiving 100 mg/m^2 ^docetaxel 3-weekly as single therapy, and 15 receiving 20 mg/m^2 ^docetaxel weekly with concurrent radiotherapy. A newly developed HPLC method was used for measuring docetaxel levels, capable of quantifying docetaxel in plasma down to the nanomolar level.

**Results:**

The HPLC method showed detectable concentrations of docetaxel in plasma even after 72 hours. In the present study we have demonstrated that median docetaxel plasma levels of 3 nM can be obtained 72 hours after a dose of 20 mg/m^2^.

**Conclusion:**

The pharmacokinetics of docetaxel is characterized by great inter-individual variability and at some time points plasma concentrations for 20 mg/m^2 ^and 100 mg/m^2 ^docetaxel were overlapping. Extrapolation of these results indicates that radio sensitizing docetaxel concentrations may be present for as long as 1 week, thus supporting the use of 20 mg/m^2 ^weekly docetaxel.

## Background

Docetaxel has been tested both as a single agent and in combination with platinum as first line treatment for NSCLC [1]. In studies with previously untreated patients with NSCLC a dose of 100 mg/m^2 ^every three weeks has been used. Neutropenia occurs in most patients and dose reduction is often required. To avoid this problem, a weekly schedule of lower dose docetaxel has been used in NSCLC, breast and prostate cancer. The doses of docetaxel used were 30–40 mg/m^2 ^either alone or in combination with platinum. This modification of the schedule, has been shown to be well tolerated and reduces the incidence of severe neutropenia while maintaining therapeutic activity [2,3].

The taxanes exert their effect by binding to the β-subunit of tubulin promoting the polymerization of tubulin into stable microtubules and inhibiting de-polymerization. In addition to mitotic arrest, taxanes have been shown to induce cell death by apoptosis both in cell cultures and in vivo tumor system [4,5].

Studies have demonstrated that taxanes are extensively metabolized in the liver. Clearance is affected in patients with abnormal hepatic function but remains unaltered in elderly patients [6]. The pharmacokinetic profile of docetaxel is characterized by substantial inter-patient variability which may have clinical implications. The area under the plasma concentration curve (AUC) during the first docetaxel course is a significant predictor of time to progression [7].

Docetaxel has a high affinity for protein binding. The binding may be as high as >95%. Only the unbound fraction is clinically active. Docetaxel induced hematologic toxicity is significantly better correlated with systemic exposure to unbound drug than to exposure to total drug [8]. Studies have shown that docetaxel is mainly bound to α_1_-acid glycoprotein (AAG), lipoproteins and albumin. AAG which is an acute phase protein, is often elevated during chronic inflammation and advanced cancer. There are great inter- individual differences in the AAG levels which might influence the pharmacokinetics of docetaxel and thereby its toxicity [7].

Docetaxel has also demonstrated activity as a radio sensitizer in a number of preclinical and clinical studies by blocking the cell cycle in the most radiosensitive G2/M phase. Combination of low dose chemotherapy and thoracic radiotherapy has been proposed to improve the prognosis in patients with locally advanced non-small cell lung cancer.

Cancer cells tested in vitro gave IC_50 _values for docetaxel ranging from 5 to 50 nM [9]. The radio sensitizing activity of taxanes in vitro however is achieved even at sub-nanomolar concentrations. Docetaxel concentrations as low as 0.07 nM has been shown to potenziate radiotherapy in cell lines [10]. An in vivo study in mice [11] showed that when docetaxel was given 9 hours before radiation, the enhancement factor was 1.45, but when radiotherapy was delayed with 48 hours, the enhancement factor was as high as 2.33. Docetaxel given within 2 days before irradiation acts as a potent enhancer of tumor radio response and increases the therapeutic gain of irradiation.

With concurrent radiation the weekly docetaxel doses used ranges from 20 to 35 mg/m^2^. In a recent publication [12] reviewing different phase I/II studies with concurrent radiotherapy and docetaxel alone or in combination with platinum compounds, a number of different doses and schedules were used. The authors were however unable to draw firm conclusions from the data to which dose and schedule was the most efficient.

In our Nordic phase III combined modality study more than 250 patients with inoperable NSCLC were randomized to radiotherapy 2 Gy × 30 alone or with concurrent chemotherapy with docetaxel 20 mg/m^2 ^weekly for 6 weeks. The same total dose of docetaxel was used as in our previous phase I/II study [13] but now as 6 weekly infusions rather than 4 (total dose of docetaxel 120 mg/m^2^), which is the same dose as recommended by Mauer et al [14] in their trial. The patients included in the 20 mg/m^2 ^group were treated according to this phase III protocol. The pharmacokinetic sampling was however not a part of this protocol, but defined as a separate study. Several reversed phase high performance liquid chromatographic methods (HPLC) have been used for detecting docetaxel. Recently a more sensitive and specific method for detecting docetaxel in nanomolar concentrations in human plasma was developed at our hospital [15].

In the present study we have used this method to monitor pharmacokinetics of low dose docetaxel up to 72 hours.

## Methods

The clinical trial was approved by the Regional Committee for Medical Research Ethics and the Hospital Review Board. The study was in compliance with the World Medical Association of Helsinki. All patients were included after having signed a written informed consent. A separate informed consent was obtained for patients participating in the pharmacogenetic analyses.

The dose of docetaxel was determined by body surface area (BSA) [16]. Docetaxel (Taxotere^® ^Sanofi Aventis, Bridgewater, NJ, USA) was administered once every week at 20 mg/m^2 ^with concurrent radiotherapy (n = 15) or as 100 mg/m^2 ^(n = 19) as single drug every 3-weeks without radiotherapy.

### Weekly regimen (20 mg/m^2^)

Patient characteristics are described in Table 1.

**Table 1:** Patient characteristics

**Docetaxel dose**	**20 mg/m**^**2**^	**100 mg/m**^**2**^
Number of patients	15	19
Sex		
Male	11	7
Female	4	12
Age, years		
Median	69	63
Range	47–81	45–75
Histological type		
Adenocarcinoma	3	12
Squamous cell	9	4
Large cell	1	2
Undifferentiated	2	1
Performance status		
ECOG 0	6	5
ECOG 1	7	10
ECOG 2	2	4
Stage		
IIIA	3	
IIIB	12	4
IV		15

Inclusion criteria: Histological or cytological confirmed locally advanced NSCLC disease where surgery was not possible. Performance status, ECOG ≤ 2. Weight loss of less than 10% during the last 6 months. Adequate hematological, pulmonary, renal and hepatic function, i.e. neutrophils > 1.5 × 10^9^/l, platelets > 100 × 10^9^/l, FEV1 > 40%, total serum bilirubin < 1.5 × upper normal limit, serum creatinine < 1.5 × upper normal limit, ASAT, ALAT, and LDH ≤ 2 × upper normal limit.

### 3-weekly regimen (100 mg/m^2^)

Patient characteristics are described in Table 1.

Inclusion criteria: Histological or cytological confirmed NSCLC with locally advanced disease not suitable for radiotherapy with curative intention, or with metastatic disease. The patients were physically fit for chemotherapy, e.g. ECOG ≤ 2. Bilirubin, ASAT, ALAT < 1.5 times upper normal limit. Predicted life expectancy ≥ 3 months.

### Patient evaluation

All patients underwent a complete medical history and physical examination, documentation of ECOG status including routine hematology and biochemistry analysis. Staging was done with chest radiography, computed tomography of the chest and upper abdomen. Patients with significant underlying medical conditions were excluded. CT or MRI examination of the brain was only performed when medically indicated.

### Treatment

#### Weekly regimen (20 mg/m^2^)

Eligible patients received weekly intravenous docetaxel 20 mg/m^2 ^for 6 courses with concurrent radiotherapy. The chest irradiation was given to a total dose of 60 Gy (2 Gy × 30) using a minimum of 3 treatment fields determined by CT scan. The radiation therapy was given 5 days each week for 6 weeks.

Docetaxel was diluted in 100 ml isotonic NaCl. Patients were pre-treated with dexamethasone 8 mg orally twice daily for two days, starting the day before the infusion. Docetaxel was administered as a one hour infusion, preferably on Monday or Tuesday, no later than on Wednesday, thus the interval between two docetaxel infusions was 144 hours. Of 25 examined courses of docetaxel 20 mg/m^2 ^given with concurrent radiotherapy, 21 were given Monday or Tuesday. On the day of infusion, radiotherapy was given within 2 hours after docetaxel infusion.

#### 3-weekly regimen: (100 mg/m^2^)

The patients were treated with docetaxel for a maximum of 6 courses. The treatment was terminated if unacceptable toxicity occurred or at disease progression. Docetaxel was diluted in 250 ml isotonic NaCl. Pre-treatment with dexamethasone as mentioned above. The infusion time for docetaxel was 1 hour. The first course of chemotherapy was given at our institution; eight patients were then referred to their local hospital for completing the chemotherapy. Eleven patients received all courses of docetaxel at our institution.

### Blood sampling

Blood samples were obtained at the following time points: pre-treatment, 30 minutes (mid-infusion), 55 minutes, 90 minutes and at 2, 3, 6, 10, 20, 25, 48 and 72 hours post infusion if possible. The samples were drawn from an intravenous catheter located in the arm opposite to the docetaxel infusion, collected in 9 ml tubes containing EDTA as anticoagulant (Vacuette, Greiner), and placed on ice until additional processing within 60 minutes. Plasma was isolated by centrifugation for 12 minutes at 1000 × g at +4°C. The plasma was frozen at -20°C after centrifugation. For 10 of 15 patients in the weekly and 5 of 19 in the 3-weekly group, we were able to obtain blood samples from 2 courses of chemotherapy. Two patients treated with 100 mg/m^2 ^were monitored for more than two cycles. A total of 52 courses have been analyzed. Due to practical reasons (most patients treated as out patients etc) we were able to take blood samples at 72 hours only from patients in the 20 mg/m^2 ^group with concurrent radiotherapy.

### Analytical procedure

Plasma docetaxel concentrations were measured over the range of 1 to 1000 nM using a validated HPLC method developed in our laboratory. The method is described in detail elsewhere [15]. The method has sufficient sensitivity to detect docetaxel in plasma up to 72 hours after an infusion of 20 mg/m^2 ^of the drug. Briefly, docetaxel was extracted from plasma using solid phase extraction on CN-E columns and separated on a reversed phase C-18 column. UV detection was performed at 227 nm, and paclitaxel was used as internal standard in the assay. Samples expected to exceed the upper limit of quantitation of the assay were prediluted in drug-free plasma according to a defined set of volume ratios.

AAG concentrations were measured immunonephelometrically on a Beckman Immage™ Immunochemistry System (Beckman Instruments) using the Beckman AAG kit.

### Pharmacokinetic calculations

Individual pharmacokinetic parameters were calculated using PK Solutions 2.0 (Summit Research Services, CO, USA). Area under the concentration-time curve (AUC_0-t_) was determined by trapezoid calculation using measured concentrations. Unless stated otherwise, AUCs given are AUC_0–25 h_. Systemic clearance [L/h/m^2^] was calculated as dose divided by AUC_0–25 h_.

### Statistical analysis

This is a small study of a descriptive nature. A statistical section has not been included as no statistical comparisons between the groups were planned in the protocol, and no statistical comparisons between the groups were included in the manuscript.

## Results

### Patient characteristics

Thirty-four patients, 18 males and 16 females with advanced or metastatic NSCLC were enrolled in this study between August 2000 and May 2006 (see Table 1). The inclusion of patients in the 3-weekly part of the study was terminated in 2002. Their median age was 63 years. The most common histological types were adenocarcinoma (15 patients) and squamous cell carcinoma (13 patients).

### Dose administration

Fourteen patients in the weekly group received docetaxel as planned (6 courses of docetaxel) during concurrent radiotherapy. One patient received 3 courses of docetaxel. We were able to administer 87/90 courses (96%) of weekly docetaxel as planned.

Nineteen patients received docetaxel 100 mg/m^2 ^every 3-weeks. According to the patients files, only four patients received the planned maximum of 6 doses of docetaxel, median 3 courses (range 1–6).

### Docetaxel pharmacokinetics

The pharmacokinetic model for docetaxel is a three-compartment model with first order elimination. Pharmacokinetic parameters for all cycles given as weekly or 3-weekly docetaxel are shown in Table 2. Overall we had 10% missing samples due to patients receiving radiotherapy at some time points or technical problems with blood sampling. Due to missing samples, not all of the pharmacokinetic parameters could be calculated for all the patients.

**Table 2:** Docetaxel pharmacokinetic parameters in patients treated with 20 mg/m^2 ^and 100 mg/m^2^

	All cycles*			
**Variables**	**20 mg/m**^**2**^		**100 mg/m**^**2**^	
	
	**Median**	**Range**	**Median**	**Range**

C_max _[nM]	742	389–1294	3737	2616–6949
AUC_0–25 _[nMh]	1284	848–1812	5562	3653–12790
AUC_0–72 _[nMh]	1541	996–2038	ND	ND
Clearance [L/h/m^2^]	19.3	14–29	22.3	10–34
t_1/2α _[h]	0.99	0.8–2.0	0.74	0.5–1.0
t_1/2β _[h]	12.9	9–35	18.0	9–52
t_1/2γ _[h]	27.7	14–146	ND	ND
C_72h _[nM]	3.0	0.7–6.5	ND	ND
AAG [g/L]	1.6	0.7–2.7	1.5	0.8–2.9

*: Each variable was calculated based on 11 to 27 cycles.

The median docetaxel clearance for all cycles (n = 25) in the weekly regimen was 19 (14–29) L/h/m^2^. Median C_max _was 742 nM. Median AUC for the first course (n = 15) was 1360 nMh with a range from 900 to 1660. For the second course (n = 10), median AUC was 1260 nMh (range 848–1812).

**Figure 1:**
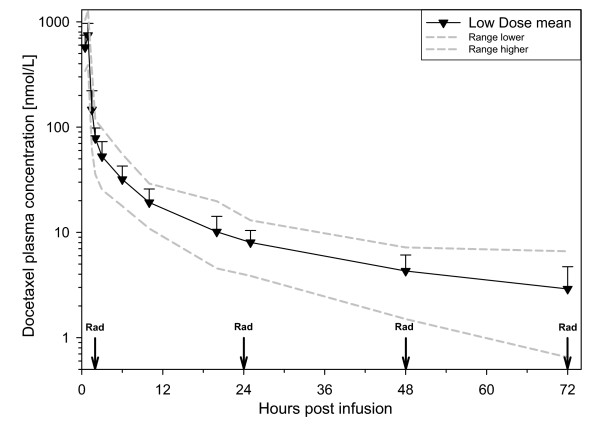
Elimination curve for patients receiving 20 mg/m^2 ^of docetaxel. Mean values (▼), with error bars representing 1SD. Gray lines represent the extreme values at each time point. Vertical arrows labelled Rad represent the time of radiation treatment.

The time schedule between radiotherapy and plasma concentration of docetaxel is illustrated in Figure 1. Docetaxel was detectable in all samples taken at 72 hours (n = 14). Median concentration 3.0 nM (0.7–6.5).

For all cycles (n = 27) in the 3-weekly group median docetaxel clearance was 22 (10–34) L/h/m^2^. Median C_max _was 3737 nM. The AUC range for the first course (n = 19) of chemotherapy was 3653–12790 nMh, median 5718 nMh. For the next course (n = 5), the range was 5183–6862 nMh and the median 5562 nMh.

The differences in intra-individual concentrations were registered for 15 patients given two or more cycles in our study (Table 3). When comparing AUC 2/AUC 1 for 10 patients in the 20 mg/m^2 ^group, the median ratio was 1.12 (range 0.6–1.5). For the 3 weekly regimen, we have samples from 5 patients, the ratio of AUC 2/AUC 1 was 1.13 (range 0.9–1.5).

**Table 3:** Intra-individual variability in docetaxel pharmacokinetic parameters in patients treated with 20 mg/m^2 ^and 100 mg/m^2^

	**Ratio cycle 2/cycle 1**^**#**^			
**Variables**	**20 mg/m**^**2**^		**100 mg/m**^**2**^	
	
	**Median**	**Range**	**Median**	**Range**

C_max_	1.06	0.4–1.6	1.07	0.9–1.3
AUC_0–25_	1.12	0.6–1.5	1.13	0.9–1.5
AUC_0–72_	1.12	0.8–1.3	ND	ND
Clearance	0.90	0.8–1.7	0.89	0.8–1.1
t_1/2α_	0.94	0.4–1.1	1.15	1.1–1.5
t_1/2β_	0.95	0.7–1.5	1.15	0.7–1.4
t_1/2γ_	1.03	0.7–1.3	ND	ND
C_72h_	0.92	0.6–2.1	ND	ND
AAG	0.98	0.7–1.7	1.15	0.9–1.4

#: Each variable was calculated based on data from 4 to 10 patients.

When analysing for AAG, the values for the weekly and the 3-weekly groups were similar: low dose median first course 1.7 (0.7–2.7), median second course 1.7 (0.7–2.1). High dose: median 1.4 (0.8–2.9) for the first course and 1.5 (1.2–1.7) for the second course.

From two of the patients in the 3-weekly group we obtained samples from three and four consecutive 3-weekly courses. The AUCs were 4800, 5500 and 4900 for cycle 1, 2 and 3 respectively in the first patient, the AAG levels were, 1.3, 1.5 and 1.5 g/L.

Also in the patient that was followed for four consecutive courses the AUCs showed minimal variation, 6600, 6000, 5800 and 6300 nMh for cycle 1, 2, 3 and 4 respectively.

Unfortunately we only obtained AAG determinations for cycle three and four in this patient, 1.5 and 1.3 g/L.

**Figure 2:**
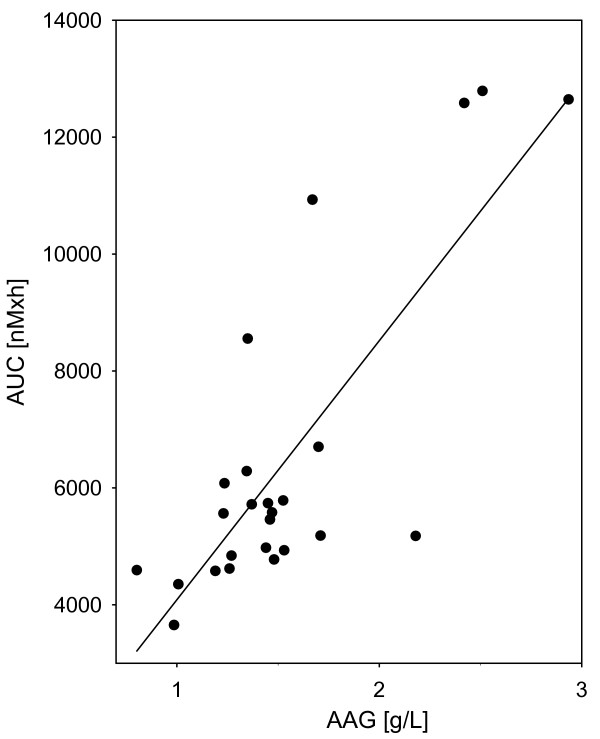
The relationship between AAG-level and docetaxel AUC for patients receiving 100 mg/m^2 ^of docetaxel (RSQ = 0.64, p < 0.0001).

**Figure 3:**
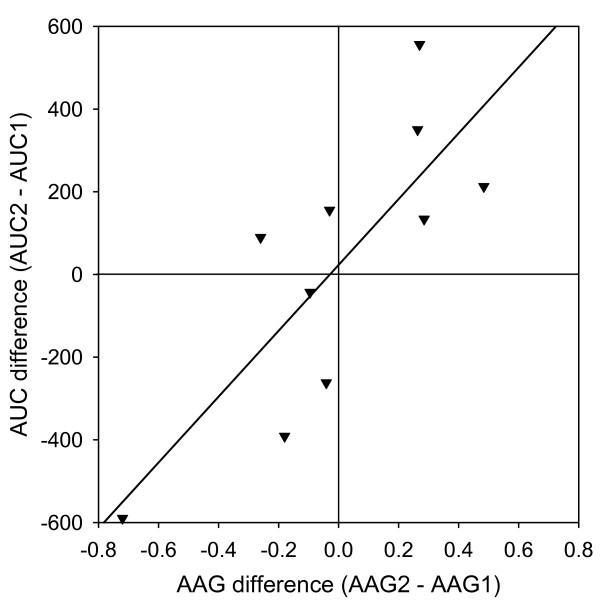
Correlation between AAG and docetaxel AUC. For the 20 mg/m^2 ^docetaxel patients that received two or more cycles, there was a correlation between the change in AAG-level and the change in docetaxel AUC_0–25 _between the cycles (RSQ = 0.63, p = 0.011).

The correlation between AUC and AAG was significant for the 100 mg/m^2 ^group of patients (Figure 2) but not for the 20 mg/m^2 ^group of patients (RSQ = 0.02, p = 0.863). For the individual patient in the 20 mg/m^2 ^group however, the difference in docetaxel AUC between the first and second course correlated with the change in AAG levels between the first and second course (Figure 3).

### Toxicity

Weekly regimen: 14/15 patients completed the treatment as planned. One patient died after 21 days due to haemoptysis after receiving 3 infusions of docetaxel. The patients were evaluated for toxicity at day 42 (end of radiotherapy) and dysphagia/oesophagitis was recorded according to CTC (Common Toxicity Criteria's version 2.0). Grade 2 or higher oesophagitis occurred in 9 of the 14 (64%) patients who fulfilled the planned treatment, and one patient (7%) had grade 3 oesophagitis. In our small group of patients 3 of 15 (20%) were reported with radiation pneumonitis. The association with AUC for docetaxel and volume of irradiated lung tissue is not clear, but all three patients had a large volume of irradiated lung tissue.

For patients in the 3-weekly regimen, registration of toxicity was mostly reported for treatment given at our institution. Except for the anticipated toxicities with fever and infection, we observed 2 patients with serious pulmonary toxicity. Two patients developed paraesthesia/neuropathy. The reasons for discontinuation were death (2 patients), progressive disease (6 patients), and reduced general condition (2 patients). Only 2 of 19 patients were alive after 1 year.

## Discussion

The purpose of this study was to investigate inter- and intra-individual variability in the pharmacokinetics of docetaxel administered as 3-weekly and weekly schedule. Weekly docetaxel is often used in concurrent chemo-radiotherapy regimens. In the present study, we also examined if radio sensitizing concentrations of docetaxel in plasma could be obtained and if the concentrations were maintained over sufficient periods of time. Docetaxel is eliminated mainly by the subgroup Cyp 3A4 and Cyp 3A5 of the cytochrome P 450 system [17].

When analyzing SNPs predictors of docetaxel clearance in a previous study, other groups of enzymes also important for docetaxel clearance were identified, e.g. GSTs, EPHX1/2, BLC2, EGF and EGFR [18].

**Figure 4:**
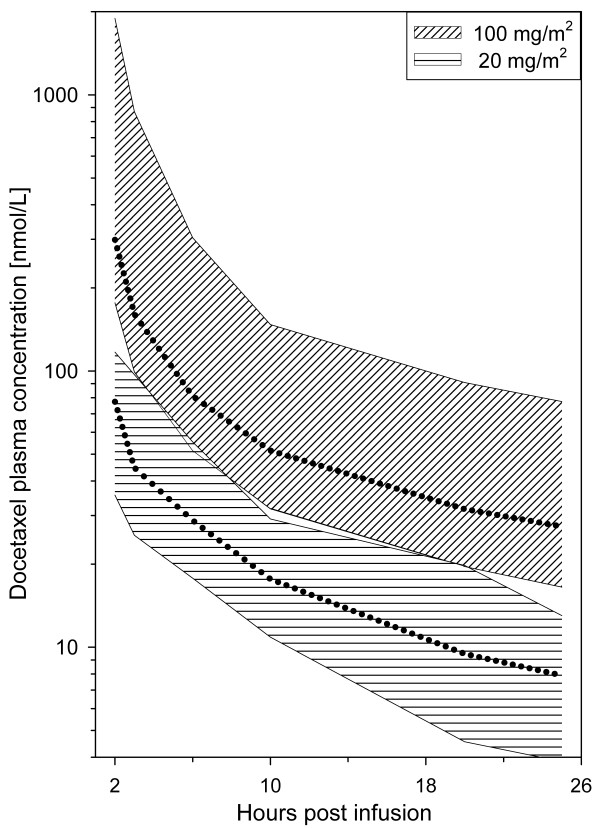
Interpatient variations for two docetaxel dose levels. Plotting the total range (dotted line = median) of docetaxel concentrations at each time point, the graphs for the patients receiving 20 mg/m^2 ^and the patients receiving 100 mg/m^2 ^will overlap at some time points (e.g. at 6 h: low dose range 18–55 nM, high dose range 51–304 nM). For simplicity, the graph shows determinations from 2 to 25 hours.

It is established that the pharmacokinetic profile of docetaxel is characterized by substantial inter-patient variability. Bruno et al [7] reported a 3.5 fold variation in clearance in 600 patients receiving 75–100 mg/m^2 ^of docetaxel. Similarly, our results showed AUC values differing 3.5 fold for the 100 mg/m^2 ^group and more than 2 fold for the 20 mg/m^2 ^group. Plasma concentrations for the two groups were even overlapping at some time points (Figure 4). Most previous pharmacokinetic data for docetaxel originates from studies with 75–100 mg/m^2 ^infused over 1 hour every third week. Pharmacokinetic data for docetaxel at such high doses follow a three compartment model with half-lives of 4 minutes, 38 minutes and 12 hours in the α, β and γ phase respectively up to 24 hours post infusion. Total body clearance is 21 L/h/m^2^, and the volume of distribution at steady state (V_ss_) is 74 L/m^2 ^[9]. In a study by Baker et al [19] comparing pharmacokinetics of weekly- and 3-weekly docetaxel, it was demonstrated that the docetaxel pharmacokinetics are similar for the two dose levels. Our results are in accordance with their findings, in our study the clearance was 22 (10–34) L/h/m^2 ^for the high dose patients and 19 (14–29) L/h/m^2 ^for the low dose patients.

Few studies have reported pharmacokinetic data from more than one course of chemotherapy for their patients. The differences in intra-individual docetaxel concentrations were registered for 15 patients. The median ratio for AUC 2/AUC 1 was close to 1 and almost identical for the 20 mg/m^2 ^and the 100 mg/m^2 ^group. The range was from 0.6 to 1.5. The intra-individual differences were smaller than the inter-individual differences, a finding which is similar to the results in the study by Engels et al [20].

Docetaxel is to a great extent bound to plasma proteins and we found that the intra-patient AUC 2/AUC 1 variability possibly could be explained by changes in AAG levels. As described by Baker [21] serum AAG levels can vary up to 7-fold between cancer patients, and in our study the variation was approximately 4-fold.

We found a statistically significant correlation between AUC and AAG for the 100 mg/m^2 ^group of patients (Figure 2) but not for the 20 mg/m^2 ^patients (RSQ = 0.02, p = 0.863). The difference in AUC between the first and second course of docetaxel in the 20 mg/m^2 ^group could partly be explained by variations in the measured AAG levels (Figure 3). High levels of AAG have been found to be associated with a low fraction of "free" docetaxel which may influence the clinical efficacy. This observed variability in AAG levels may also complicate the interpretation of pharmacokinetic results when based only on determinations of total drug concentrations.

Bruno reported that in patients in the 100 mg/m^2^, docetaxel exposure measured as AUC during the first course of chemotherapy was the only significant predictor of toxicity [22]. In our study 4 of the patients had AUC levels above 10000. However, the patient with the highest AUC level developed serious respiratory complications, while for the other three no specific complications were noted.

The use of docetaxel as a radiosensitizer was well tolerated, and all patients received treatment as planned except for the patient that died from haemoptysis. The incidence of grade 3 oesophagitis was 7% which is lower than reported by Mauer [14] and Scagliotti [23].

The question has been raised if weekly docetaxel in 20 mg/m^2 ^dose, used in concurrent chemo-radiotherapy regimens, will give sufficient plasma levels over time to have radio-sensitizing activity. A study by Pradier et al found concentrations as low as 0.7 and 0.07 nM to potenziate radiotherapy in two different cell lines [10]. Also Choy [24], demonstrated that such low nano-molar concentrations still enhance radio-sensitivity. Even in cell lines that rarely responds to radiation or chemotherapy, docetaxel concentrations of 0.3–1 nM was found to have a radio-sensitizing potential [25].

There are few reports of the corresponding relationship between plasma concentrations and the actual concentration in the tumor. In a study with paclitaxel in patients with uterine cervical cancer, Mori et al [26] demonstrated that paclitaxel was still retained in cancer cells for 1 week in vivo and in vitro, thus supporting the use of weekly regimens of paclitaxel.

Examining the radiobiological effects of docetaxel as a radio sensitizer, Creane [27] found no increase in radio sensitizing efficacy when docetaxel concentration was raised from 4–10 nM while duration of the exposure was more important than the actual drug concentration in the tumor.

Baker et al [19] have demonstrated that docetaxel concentrations could be maintained above 1 nM for 7 days with weekly schedules and above 0.5 nM for 21 days when given with 3-weekly regimens. They analyzed docetaxel concentrations for up to 21 days, and concluded that the circulation time of docetaxel in cancer patients has been greatly underestimated. In a study by Gustafson [28] docetaxel concentrations after 30 mg/m^2 ^for up to 48 hours was obtained. Extrapolating the results they found it likely that plasma levels above 1 nM would be present at 68 hours with this dosing. In our study, analyzing the plasma levels at 72 hours, we could confirm that docetaxel levels above 1 nM (median 3 nM) could be maintained as long as 72 hours with 20 mg/m^2 ^dose. Extrapolating our 72 hours results using a half-life of 60 hours [19], suggest that the median docetaxel concentration was 1–2 nM when the last radiation treatment in each weekly cycle was given. In a recent publication by Huber [29], it could be shown that weekly paclitaxel with concurrent radiotherapy was superior to radiotherapy alone. Whether weekly docetaxel with concurrent radiotherapy is also more effective than radiotherapy alone, is being addressed in the aforementioned Nordic randomized phase III trial recently closed for inclusion.

## Conclusion

The present study demonstrates significant interpatient differences in docetaxel pharmacokinetics. The differences can partly be explained by differences in AAG levels, but AAG levels may change within a week as measured in our study. For the first time it was demonstrated that after weekly regimen of docetaxel 20 mg/m^2^, docetaxel concentrations above 1 nM was present in plasma after 3 days. Extrapolating the results, it can be estimated that radio-sensitizing docetaxel levels still are maintained in plasma after 7 days, thus providing a rationale for the use of weekly docetaxel 20 mg/m^2 ^and concurrent radiotherapy.

## Competing interests

The author(s) declare that they have no competing interests.

## Authors' contributions

PB, AA, SAA and HO have designed the study. PB has drafted and written the manuscript together with AA, SAA and HO. AA has performed the laboratory work. PB and SA have been responsible for the clinical part of the study. VK have analyzed the pharmacogenetics and revised the final manuscript.

## Pre-publication history

The pre-publication history for this paper can be accessed here:


